# Three-dimensional HepaRG spheroids as a liver model to study human genotoxicity *in vitro* with the single cell gel electrophoresis assay

**DOI:** 10.1038/s41598-019-47114-7

**Published:** 2019-07-22

**Authors:** Marion Mandon, Sylvie Huet, Estelle Dubreil, Valérie Fessard, Ludovic Le Hégarat

**Affiliations:** 1 ANSES, French Agency for Food, Environmental and Occupational Health and Safety, Fougères Laboratory, Toxicology of contaminants unit, 10 B rue Claude Bourgelat, Fougères, 35306 France; 2 ANSES, French Agency for Food, Environmental and Occupational Health and Safety, Fougères Laboratory, Analysis of residues and contaminants unit, 10 B rue Claude Bourgelat, Fougères, 35306 France

**Keywords:** Cancer, Drug discovery

## Abstract

Many efforts have been made in the last 30 years to develop more relevant *in vitro* models to study genotoxic responses of drugs and environmental contaminants. While 2D HepaRG cells are one of the most promising models for liver toxicology, a switch to 3D cultures that integrate both *in vivo* architecture and cell-cell interactions has occurred to achieve even more predictive models. Preliminary studies have indicated that 3D HepaRG cells are suitable for liver toxicity screening. Our study aimed to evaluate the response of HepaRG spheroids exposed to various genotoxic compounds using the single cell gel electrophoresis assay. HepaRG spheroids were used at 10 days after seeding and exposed for 24 and 48 hours to certain selected chemical compounds (methylmethansulfonate (MMS), etoposide, benzo[a]pyrene (B[a]P), cyclophosphamide (CPA), 7,12-dimethylbenz[a]anthracene (DMBA), 2-acetylaminofluorene (2-AAF), 4-nitroquinoline (4-NQO), 2-amino-1-methyl-6-phenylimidazo[4,5-b]pyridine (PhIP), 2-amino-3-methylimidazo[4,5-f]quinolone (IQ), acrylamide, and 2-4-diaminotoluene (2,4-DAT)). After treatment, the comet assay was performed on single cell suspensions and cytotoxicity was determined by the ATP assay. Comet formation was observed for all compounds except IQ, etoposide and 2,4-DAT. Treatment of spheroids with rifampicin increased CYP3A4 activity, demonstrating the metabolic capacity of HepaRG spheroids. These data on genotoxicity in 3D HepaRG spheroids are promising, but further experiments are required to prove that this model can improve the predictivity of *in vitro* models to detect human carcinogens.

## Introduction

Information on genetic toxicity is an essential part of the safety assessment for any type of substance. In the absence of clear carcinogenic data, genotoxic studies may be useful to conclude on the carcinogenic potential of chemical substances. To align with what occurs in humans, *in vitro* models that closely reflect the structural and functional characteristics of human tissues are being developed. Although *in vivo* studies can investigate the genotoxic effects of compounds in different tissues in animals, there are still differences between animals and humans, including in the genetic and metabolic systems, which can lead to inconsistent conclusions^1^.

Considering that most of genotoxic carcinogens in humans require metabolic activation, *in vitro* cell models need to mimic human metabolism as closely as possible, especially concerning the liver. Usually, metabolic activation is investigated through the addition of an induced rat liver S9 fraction, containing major cytochrome P450 isoforms and other metabolic enzymes, during *in vitro* incubations with the compounds^2^. However, although relevant to elucidate the mode of action of a carcinogenic substance in rodents, the use of a rat liver fraction remains questionable to predict mutagenicity in humans, considering species-specific metabolic characteristics. Therefore, the development of alternative models that could improve the predictivity of *in vitro* genotoxicity tests to detect human carcinogens without using a rat liver fraction is a challenge for hazard assessment.

Although the liver is a key organ for xenobiotic detoxification, it is also involved in the bioactivation of various human carcinogens. Genotoxic metabolites can be formed following one or more steps of phase I and/or II metabolism. Therefore, human liver cell models that are metabolically competent are required for *in vitro* genotoxicity testing. To study drug metabolism and toxicity, primary human hepatocytes (PHH) are currently considered the gold standard *in vitro* model^3^. However, several disadvantages with PHH, including limited availability, inter-individual variability, and early dedifferentiation to progenitor-like cells lacking relevant hepatic gene expression, have been reported^1,4^. Consequently, immortalized cell lines are commonly used^5^. Although widely used for toxicity studies, the HepG2 hepatoma cell line presents low activities of several drug-metabolizing enzymes, especially some cytochrome P450 isoforms^6^. The well-characterized HepaRG cell line in 2D culture depicts a closer phenotype to PHH than HepG2 cells concerning the expression of phase I and II enzymes, transporters, and nuclear factors. This phenotype is stable in culture for several weeks and responds to various inducers^7–9^. Moreover, this model was shown to be suitable to detect genotoxic compounds with various tests: comet assay, micronucleus, γH2AX, as well as genotoxicity-targeted qPCR array^2,10–14^. However, some promutagen compounds have remained difficult to detect in 2D HepaRG cells, such as styrene and the aromatic amines IQ, MeIQX and 2,4-DAT, possibly due to low CYP2E1 and CYP1A2 activities and N-acetyl-, or sulfotransferase (NAT, SULT) activities^2,13,15^.

Although hepatic 2D cell cultures have been widely used to predict *in vivo* responses to chemicals, they do not mimic the complexity of human tissues *in vivo*^16^. In fact, 2D liver cell models may have abnormal gene expression profiles, and do not share the 3D structure of the liver^17,18^. To address these limitations, 3D models that promote cell-cell and cell-extracellular matrix interactions have been developed and are thus considered more relevant to predict *in vivo* responses. Currently, several 3D *in vitro* human hepatic systems are available such as matrices and scaffolds, bioreactors, and microfluidic cell culture platforms^19^. However, these systems are technically challenging, labor intensive, and expensive, and are not suitable for high-throughput applications^20^. Another method, 3D spheroid culture, requires few cells, is easy to handle, and is appropriate for high-throughput studies^21,22^.

HepG2 spheroids have been reported as a promising model to study *in vitro* genotoxicity, providing better results than 2D monolayer cultures^23^. In 2017, the development of HepaRG spheroid cultures in ultra-low attachment plates that support spheroidal differentiation was described^24^. Spheroids maintained a stable phenotype, exhibiting several hallmarks of polarized hepatocyte architecture and functions, including metabolizing enzyme activities, long-term stable albumin secretion, and transporter localization^20,25^.Authors showed that 3D HepaRG cells express the three major P450 enzymes (CYP1A2, CYP2B6 and CYP3A4) and active hepatic nuclear receptors (AhR, CAR and PXR)^24^. As this model probably presents the ability to bioactivate xenobiotics due to high levels of CYP enzyme activities maintained for up to 28 days, genotoxicity assays on HepaRG spheroids are worth developing.

Considering that (i) 2D HepaRG cells have failed to detect some progenotoxic compounds, (ii) 3D HepaRG spheroids have shown metabolic activities and functional characteristics that are closer to those of the human liver, and (iii) results have shown that the 3D HepG2 model is more sensitive than the 2D model to detect human mutagens, we proposed to evaluate the response of HepaRG spheroids exposed to various genotoxic compounds using the single cell gel electrophoresis assay (comet assay). We studied the sensitivity of HepaRG spheroids to both genotoxicants and pro-genotoxicants, and compared these results to those obtained with 2D differentiated HepaRG cells.

## Materials and Methods

### Chemicals

2-acetylaminofluorene (2-AAF), 2,4-diaminotoluene (2,4-DAT), 4-nitroquinoline (4-NQO), 7,12-dimethylbenzaanthracene (DMBA), acrylamide (AA), cyclophosphamide (CPA), dimethylsulfoxide (DMSO), etoposide, methylmethanesulfonate (MMS), omeprazole (OME) phenobarbital (PB), rifampicin (RIF), phenacetin, diclofenac, bupropion, midazolam, acetaminophen, 4′-OH-diclofenac, 1′-OH-midazolam, OH-bupropion, diclofenac-d_4_ (internal standard), and acetaminophen-d_4_ (internal standard) were purchased from Sigma (Saint-Quentin-Fallavier, France). 2-amino-1-methyl-6-phenylimidazo[4-5b]pyridine (PhIP) and 2-amino-3-methylimidazo[4-5f]quinolone (IQ) were obtained from Toronto Chemical Research (Toronto, ON, Canada).

### Preparation of the tested compounds

For the comet assay, 11 chemicals were selected. MMS and CPA were dissolved in FCS-free medium, 4-NQO, etoposide, AA, 2,4-DAT, DMBA, 2-AAF, PhIP, IQ and B[a]P were dissolved in DMSO. For all assays, the final concentration of DMSO never exceeded 0.5%. For CYP induction, OME, PB and RIF were dissolved in DMSO.

### HepaRG cell culture

Cells were cultured in Williams E medium (Eurobio, Les Ulis, France) supplemented with 10% FCS (Perbio, Brebières, France), 100 units/mL penicillin (Invitrogen Corporation, Illkirch, France), 100 µg/mL streptomycin (Invitrogen Corporation), 5 µg/mL insulin (Sigma-Aldrich, Lyon, France), 2 mM L-glutamine (Thermofisher, Waltham, MA, USA), and 25 µg/mL hydrocortisone succinate (Pharmacia & Upjohn, Guyancourt, France).

### 3D HepaRG spheroid formation

**Figure 1:**
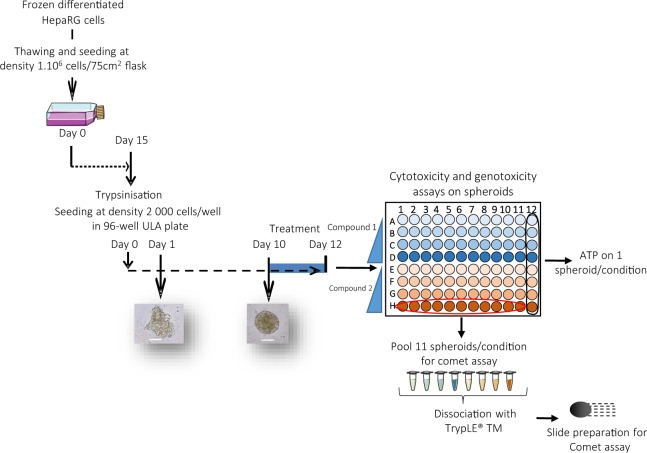
3D HepaRG spheroid culture and treatment schedule for the ATP and comet assays.

1.10^6^ HepaRG cells were seeded in a 75 cm^2^ flask and were incubated at 37 °C with 5% CO_2_ and medium was changed every 2 days (Fig. 1). After 14 days, cells were trypsinized prior to dissociation with a syringe to obtain a suspension of isolated cells. Then, cells were seeded in 96-well ultra-low attachment (ULA) plates (Corning, Boulogne-Billancourt, France) at a density of 2,000 cells/well. Medium was changed after 7 days and spheroids were used at Day 10.

### Comet assay

After 24 or 48 hrs of treatment, medium was removed and spheroids were washed twice with PBS. For each condition, 11 spheroids were pooled in a 2 mL Eppendorf. After 5 min of sedimentation, PBS was removed and 200 µL of TrypLE^TM^ without red phenol (Gibco, Courtaboeuf, France) at 37 °C was added. After 40 min of incubation at 37 °C, the dissociation of spheroids was checked under light microscope and cells were centrifuged for 5 min at 400 *g*. TrypLE^TM^ was removed and cell pellets were suspended in a pre-warmed low melting point agarose (0.5% in PBS) and deposited on conventional microscope slides (initially dipped in 1% agarose and dried) as previously described^13^. The slides were put in a lysis solution (NaCl 2.5 M, EDTA 0.1 M, Tris-HCl 10 mM, with extemporaneous addition of DMSO 10% and Triton X-100 1% at pH 10) for 1 hr at 4 °C. DNA was allowed to unwind for 40 min in electrophoresis buffer (NaOH 0.3 M, EDTA 1 mM, pH 13) prior to electrophoretic migration (0.7 V/cm, 300 mA) for 24 min at room temperature. The slides were incubated twice in neutralizing solution (Tris-HCl 0.4 M, pH 7.5) for 5 min and dried for storage with 95% ethanol for 5 min. DNA was stained with propidium iodide (2.5 µg/mL in PBS) just before examining the slide with a fluorescence microscope (Leica DMR) equipped with a CCD-200E video camera. At least two slides per concentration and 100 cells per slide were analyzed using Comet Assay IV software (Perceptive Instruments, Haverhill, UK). The percentage of DNA in the comet tail (% tail DNA) was used to evaluate the extent of DNA damage. At least three independent experiments were conducted.

### Cytotoxicity assay

For each treatment condition, one spheroid was used for the cytotoxicity assay. The assay was performed using a CellTiter Glo 3D kit (Promega, Charbonnières-les-Bains, France), according to the manufacturer’s instructions. ATP luminescence was measured using a Fluostar Optima Microplate reader (BMG Labtech, Champigny-sur-Marne, France).

### Evaluation of CYPs activities

CYPs activities were determined as previously published^26^. Briefly, for CYP induction, spheroids were incubated with PB (250 µM), RIF (2.5 µM) or OME (50 µM) for 72 hrs, with medium renewal each day. After exposure to the genotoxicants, 24 spheroids were pooled and incubated for 4 hrs with a cocktail of four substrates: 200 µM phenacetin (CYP1A2), 100 µM bupropion (CYP2B6), 100 µM diclofenac (CYP2C9), and 5 µM midazolam (CYP3A4). At the end of this period, 200 µL of medium were mixed with 200 µL acetonitrile and samples were stored at −80 °C until analysis.

### LC-HRMS conditions

LC-HRMS analysis were performed using an ultra-high pressure liquid chromatography (ThermoFisher Scientific, Bremen, Germany) system coupled with an LTQ-Orbitrap XL mass spectrometer (ThermoFisher Scientific, Bremen, Germany). Data were acquired and processed with the Xcalibur software, version 2.1 (ThermoFisher Scientific). The chromatographic column to separate correctly the metabolites was an RX-C8 Zorbax column (2.1 mm, 150 mm, 5 μm, Agilent, Les Ulis, France), protected by a C8 security guard system (12.5 mm, 2.1 mm, 5 μm). The flow rate was set at 0.25 mL.min^−1^. The column was maintained at a temperature of 25 °C in the oven and the samples were refrigerated at 10 °C inside the autosampler. Chromatographic separation was carried out applying a gradient between two mobile phase consisting of mobile phase (A): water with 0.1% formic acid, and mobile phase (B): pure analytical grade acetonitrile with 0.1% formic acid. The gradient conditions were as follows: from 0 to 6 min ramp linearly from 98 to 10% of mobile phase A, then ramp over 0.1 min to initial conditions, and hold for 6 min to re-equilibrate the system. The injection volume was set at 20 μL. The mass spectrometer was operated in electrospray positive ionization mode using the following source parameters: capillary voltage 35 V, ion spray voltage 4.5 kV, tube lens 90 V, capillary temperature 350.0 °C, sheath gas (nitrogen) flow rate 55 (arbitrary units), auxiliary gas (nitrogen) flow rate 10 (arbitrary units), sweep gas flow rate 2 units (arbitrary units). The mass spectrometer instrument was calibrated weekly using a calibration solution composed of three mass calibrators to reach mass accuracy below 5 ppm. The instrument method was run in full scan mode (FTMS) from m/z 50–800 at a resolving power of 60,000 full width at half maximum (FWHM). The accurate masses of metabolites were theoretically calculated for their [M + H]^+^ species and monitored in samples at: m/z 152.0706 for acetaminophen, m/z 256.10999 for OH-bupropion, m/z 342.0804 for OH-midazolam, m/z 312.0189 for 4′-OH-diclofenac, m/z 156.0957 for acetaminophen-d_4_, and m/z 300.0491 for diclofenac-d_4_, with a mass tolerance of 5 ppm for quantification purposes.

### Statistics

All analyses were performed using GraphPad Prism Software (GraphPad Software, Inc., La Jolla, CA, USA). Results (medians) of the comet assay from at least three independent experiments were analysed with one-way Anova followed by Dunnett’s test. Significance was established as *p* < 0.05.

## Results

### 3D HepaRG spheroid formation

**Figure 2:**
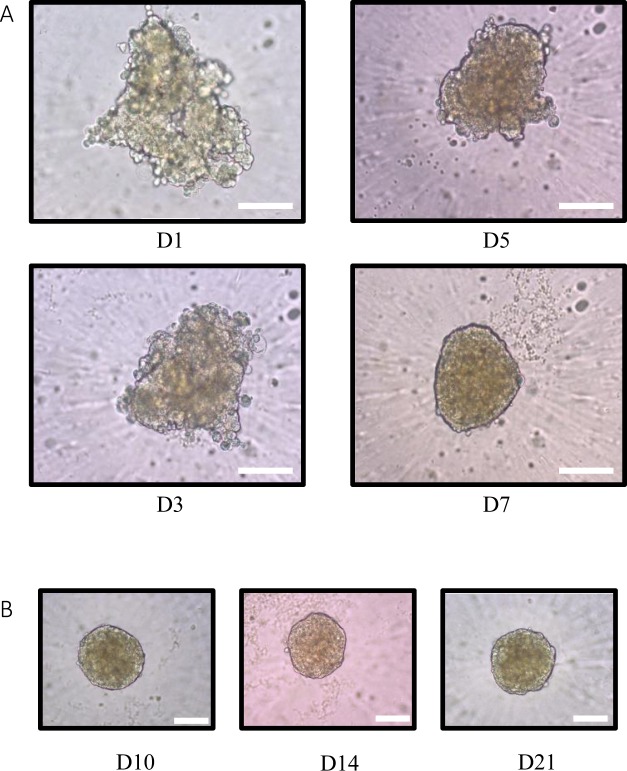
3D HepaRG spheroid. (**A**) HepaRG cells seeded at 2,000 cells per well in ULA 96-well plates. The spheroid-like structure was formed in 7 days. Scale bar = 100 µm. (**B**) Stability of size and morphology of spheroids from Day 10 to Day 21. Scale bar = 100 µm.

HepaRG cells seeded at 2,000 cells/per well in 96-well ULA plates aggregated from Day 1 to Day 3 and formed a compact spheroid structure at Day 7 (Fig. 2A). The spheroid diameter was around 100 µm and remained unchanged later on. The shape of the spheroids did not change between Day 7 and Day 21 (Fig. 2B).

### CYP activities in 3D HepaRG spheroids

CYP1A2 (100 µM phenacetin), CYP2B6 (100 µM bupropion), CYP2C9 (100 µM diclofenac) and CYP3A4 (5 µM midazolam) activities were measured with and without induction treatment by OME, PB and RIF (Table 1). No CYP1A2 activity was detected in 3D HepaRG spheroids, perhaps due to a lack of sensitivity of the LC-HRMS method used. CYP2B6 activity was detected, above the LOD but below the LOQ (0.5 µM), and LOD could be estimated at 1/3 of the LOQ, corresponding to 0.1 pmol/min-million cells. CYP2C9 activity was detected by the formation of OH-diclofenac at a rate of 1.39 ± 0.54 pmol/min-million cells. Similarly, CYP3A4 activity was detected with a formation rate of OH-midazolam at 0.41 ± 0.19 pmol/min-million cells.

**Table 1:** CYP450 activities in 3D HepaRG spheroids.

CYP	Substrate	Metabolite	CYP activity (pmol/min-million cells)			
			Control	OME	PB	RIF
CYP1A2	Phenacetin	Acetaminophen	ND	ND	ND	ND
CYP2B6	Bupropion	OH-bupropion	<LOQ^a^	<LOQ^b^	<LOQ^b^	<LOQ^b^
CYP2C9	Diclofenac	OH-diclofenac	1.39 ± 0.54	1.27 ± 0.46	2.66 ± 2.03	2.38 ± 1.79*
CYP3A4	Midazolam	1′OH-midazolam	0.41 ± 0.19	0.08 ± 0.06	1.35 ± 0.96	1.93 ± 1.54

ND: not detected, <LOD corresponding to 1/3 of LOQ (Limit of Quantification (µM)), ^a^0.5 µM corresponding to 17.36 pmol/min-million cells; ^b^0.01 µM corresponding to 0.35 pmol/min-million cells. OME = omeprazole, PB = phenobarbital, RIF = rifampicin, *p < 0.05.

Following incubation of 3D HepaRG spheroids with CYP inducers PB or RIF, some CYP activities were increased compared to solvent control. A statistically significant increase of CYP2C9 and 3A4 activities by PB was not found due to heterogeneous responses from one experiment to the other. RIF increased CYP3A4 activity in a statistically significant manner (Table 1). With OME, no CYP induction was observed and instead CYP3A4 activity was decreased compared to control.

### Cytotoxicity assay

**Figure 3:**
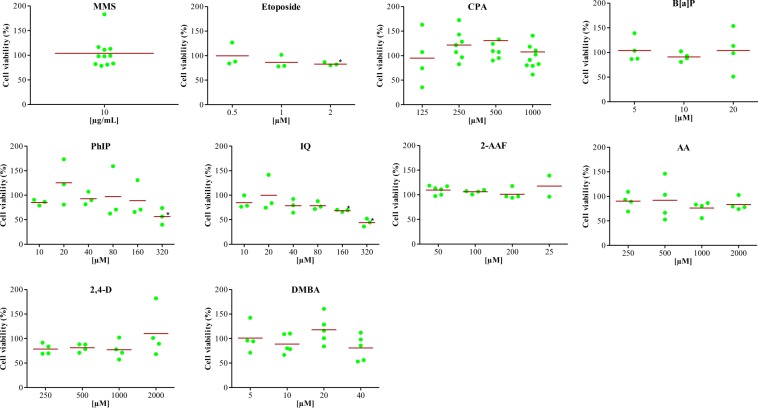
Cytotoxicity assay. Percentage of cell viability (ratio compared to negative control). Results were calculated from at least 3 independent experiments. *p < 0.05 (t-test).

For all tested compounds, cytotoxicity was assessed by ATP luminescence measurement (Fig. 3). After treatment, one spheroid was used to determine the cytotoxicity of each compound.

No cytotoxicity was found, except for IQ (at 160 and 320 µM, with 68.44% ± 1.281 and 44.15% ± 4.625 cell viability, respectively), etoposide (2 µM, 82.90% ± 2.03 cell viability), and PhIP (320 µM, 56.61% ± 9.810 cell viability).

### Comet assay

**Figure 4:**
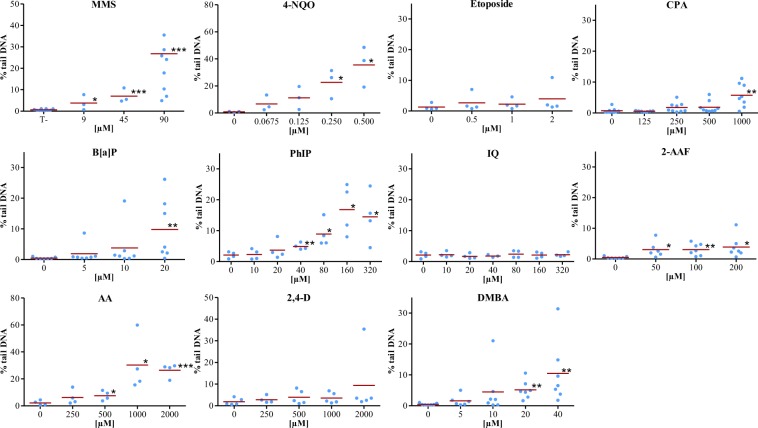
Comet assay in 3D HepaRG spheroids. Level of DNA damage measured for 11 chemicals in 3D HepaRG spheroids with the comet assay. Blue point: % of tail DNA intensity (median value obtained in each experiment); red line: mean of medians of tail intensity. Results were calculated from at least 3 independent experiments. *p < 0.05; **p < 0.01; ***p < 0.001 (t-test).

The level of DNA damage detected with solvent controls was low, between 0.24 to 2.80% tail DNA in 10 different experiments (Fig. 4).

Eleven compounds were tested by the comet assay on 3D HepaRG spheroids (Fig. 4). After 24 hrs exposure, MMS and 4-NQO increased the percentage of tail DNA in a concentration-dependent manner with a statistically significant difference at 45 and 0.25 µM, respectively. Etoposide failed to induce DNA damage in spheroids after 24 hrs of treatment up to 2 µM.

The pro-genotoxicants B[a]P, CPA, PhIP, AA, DMBA and 2-AAF significantly induced DNA damage in HepaRG spheroids at 20, 1000, 40, 500, 20 and 50 µM, respectively.

IQ failed to induce DNA damage, even at a cytotoxic concentration (320 µM). No significant differences in the percentage of tail DNA were found between the control (1.28% ± 0.51) and etoposide (3.94% ± 2.32 for the highest concentration) or 2,4-DAT (4.13% ± 1.25 for the highest concentration).

**Figure 5:**
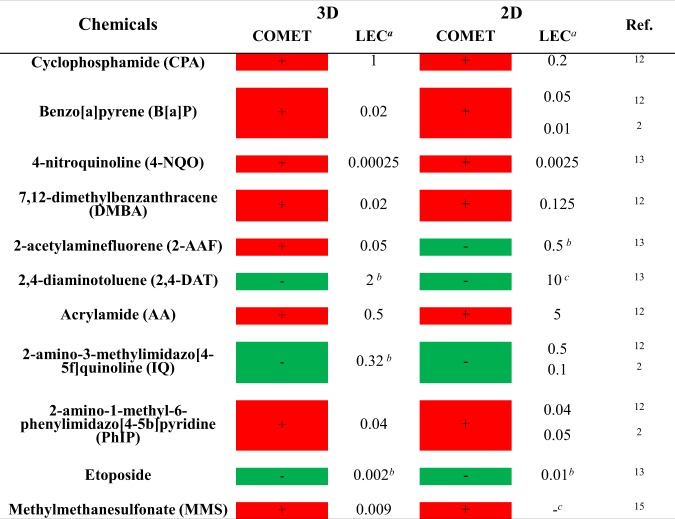
Comparison of comet assay results obtained with 3D and 2D HepaRG cells. −:Negative result; +:positive result; LEC: lowest effecting concentration (mM) yielding a positive result in the assay, b highest concentration tested, c LEC was not determined in this model.

The genotoxicity of these compounds was previously studied in a HepaRG 2D monolayer culture^2,12,13^. The results of the comet assays in both 2D and 3D HepaRG models are compared in Fig. 5. For most compounds, the results were similar in the two models, although the Lowest Observed Effecting Concentrations (LECs) of the % of tail intensities were always lower on 3D than on 2D models. A discrepant result was observed only for 2-AAF which was negative in 2D HepaRG cells but positive in 3D, even for the lowest concentration (0.48% ± 0.12 for the control, 3.03% ± 1.04 for 50 µM 2-AAF). IQ and etoposide were negative in the two models.

## Discussion

The objective of our study was to adapt the *in vitro* comet assay for use with 3D HepaRG spheroids and to determine the capacity of this new 3D model to predict *in vivo* genotoxicity. Our data demonstrate that the comet assay can be performed easily on HepaRG spheroids, and that this 3D model seems suitable to detect the majority of pro-genotoxic compounds.

The formation of HepaRG spheroids has previously been described using several different methods: hanging drops^20^, bioreactors^25^, functional polymers^27^, and ULA plates^24^. Among these strategies, spheroids formatted in ULA plates were found to be easier to handle and provided reproducible results^24^. In this previous study, 1,000 to 2,000 HepaRG cells gave a consistent spheroid size (approximately 150–200 µm), while cell numbers greater than 2,000 cells formed spheroid structures with a necrotic core and low metabolizing enzyme activities. On the basis of findings reported in the literature, we chose to seed 2,000 cells/well, providing spheroids around 100–150 µm in size without a necrotic core, contrary to HepG2 spheroids that expand rapidly, with a resulting increasing in spheroid diameter^28^.

Contrary to 2D HepaRG cells that require DMSO to stably express hepatic metabolizing enzymes, 3D HepaRG spheroids are cultured free of DMSO but still have enhanced expression of hepatic functional parameters^4,24,29^. To verify the metabolic competence of HepaRG spheroids in our study, we measured CYP enzyme activities through the formation rates of metabolites from phenacetin (CYP1A2), bupropion (CYP2B6), diclofenac (CYP2C9) and midazolam (CYP3A4), with and without PB and RIF induction. CYP1A2 could not be detected in our conditions, even after incubation with CYP inducers, probably due to the low sensitivity of our method. In fact, the LOD of acetaminophen was established at 5.8 pmol/min-million cell and the LOQ at 17.36 pmol/min-million cells, whereas another study estimated CYP1A2 activity at 12.9 pmol/min-million cells in 3D HepaRG spheroids^24^. Activity of CYP2B6 at 15 pmol/min-million cells was described in 3D HepaRG cells through the formation rate of 1-hydroxybupropion^24^, whereas we detected lower activity (only 0.1 pmol/min-million cells) in our study. These discrepancies for CYP1A2 and 2B6 activities between the two studies could be explained by the fact that we determined CYP activities at Day 10 instead of Day 21 as described by Ramaiahgari *et al*.^23^. The CYP3A4 and CYP2C9 activities were quantified by the formation of 1-OH-midazolam and OH-diclofenac, respectively, and a clear increase was observed following PB and RIF induction. Such responses suggest the presence of active hepatic nuclear receptors, as previously shown in HepaRG spheroids^24^.

Our data show that the comet assay can be adapted to 3D HepaRG spheroids. Dissociation from spheroids to individual cells is a key step providing good results with no cytotoxicity and a low level of DNA damage when using TrypLE^TM^. Positive results were obtained with the direct genotoxic compounds MMS and 4-NQO after 24 hrs of treatment.

Moreover, 3D HepaRG spheroids were able to metabolize some pro-genotoxicants into genotoxic metabolites, giving positive results in the comet assay. CPA, B[a]P, PhIP, 2-AAF, AA and DMBA induced DNA fragmentation on HepaRG spheroids after 48 hrs of treatment. Only IQ and 2-4D showed negative results in our study. Compared to the data published for 2D HepaRG cells^2,12,13,15^, similar results were obtained with HepaRG spheroids, except for 2-AAF that showed positive results in the comet assay only on the 3D model. However, although no DNA damage was detected by the comet assay with 2-AAF on 2D HepaRG cells, the formation of a micronucleus was increased statistically^13^. HepaRG spheroids seem to be more sensitive to the genotoxic effects of AA with a clear concentration increase of DNA damage up to 0.5 mM after 48 hrs of treatment. In 2D HepaRG cells, AA was only positive at the highest concentration (5 mM) after 24 hrs of treatment. These results suggest that CYP2E1 activity was higher in 3D versus 2D HepaRG models, as previously shown^20^. Other compounds such as styrene and nitrosamines should be tested on HepaRG spheroids to confirm the ability of this 3D model to detect CYP2E1-bioactivated genotoxicants.

We failed to observe genotoxicity with 2,4-DAT on the 3D HepaRG model, as previously found on 2D HepaRG cells^13^, suggesting the absence of or a low level of active N-acetyltransferase (NAT) implicated in the activation of aromatic amines like IQ, 2-AAF, and 2,4-DAT. A recent study showed that IQ required CYP1A2 in combination with NAT2, whereas SULT1A1 did not enhance its genotoxicity^30^. Nevertheless, as a positive result was obtained with 2-AAF, we expect that the 3D HepaRG model possesses at least some NAT activities. Importantly, in the comet assay, positive results with 2-AAF were observed on 3 hepatic cell lines (HepG2, HCC1.2, and Huh6), whereas IQ was negative in the same cell lines as well as negative in the micronucleus test on HepG2 cells^2,31^, but positive in Huh6 cells^32^. The genotoxic mechanism of action of IQ *in vitro* remains unclear: the link between the positive response in the comet assay and the formation of DNA-reactive nitrenium ions is hypothetic^31^. Moreover, DNA damage observed *in vitro* with IQ was probably due to oxidative stress. In fact, DNA damage was observed in primary human lymphocytes in the comet assay after 2 hrs of treatment without any metabolic activation system, suggesting that IQ induced DNA damage through oxidative stress^33^. However, HepaRG cells are less sensitive to DNA damage induced by oxidative stress than other hepatic cell lines due to high expression of antioxidant enzymes^2,4,34^.

In the present study, etoposide ETO did not affect the percentage of tail DNA, like results obtained with 2D HepaRG cultures^13^. These results suggest a low level of DNA topoisomerase II in quiescent HepaRG cells. Topoisomerase II enzyme is usually present at high levels in fast-growing cells (e.g. cancer cells) and is particularly important for DNA replication^35^. The negative response of etoposide ETO in the comet assay both with 3D and 2D HepaRG cells suggests that topoisomerase II is inactive in these cells, corresponding to the quiescent state of differentiated cells. However, etoposide ETO was shown to be genotoxic using the highly predictive qPCR array (microarray prediction of 0.97) on 2D HepaRG cells. This result was obtained on a selection of 84 genes after 72 hrs of treatment with 30 µM ETO every day^10^. The discrepancy of the results between the two studies could be explained by the higher concentration and the longer time of exposure used in the later, as treatment for more than 24 hrs could affect the cell cycle of HepaRG cells. Further experiments with others DNA topoisomerase inhibitors are required to determine the capacity of this 3D model to detect this kind of compounds.

The cellular response to pro-genotoxic compounds observed depends on the capacity of the cell model to bioactivate, detoxify and repair DNA damage, and also on the relevance of the genotoxicity test chosen. If we compare the LECs obtained with the comet assay in 3D and 2D HepaRG cells, we find that the LECs are lower with the 3D than the 2D cell model for the majority of compounds, especially for AA, DMBA and 2-AAF. However, conclusions based on LECs should be drawn with caution because the treatment conditions differed between the 2D and 3D models. In fact, only one spheroid of 2,000 HepaRG cells was exposed for 48 hrs to 100 µL of the chemical dilutions in 96-well plates, whereas 2D HepaRG cells were seeded in 48-well plates at 210,000 cells/well and exposed for 24 hrs to 200 µL of the chemical dilutions. Then, the amount of chemical per cell was 50 times higher in 3D conditions than in 2D. Therefore, the comparison of the results between the 2D and 3D models should also include the clearances of the chemicals in each condition.

The main objective of our study was to investigate the feasibility of the comet assay on 3D HepaRG spheroids to improve the *in vitro* detection of human genotoxic compounds. Taken together, the data obtained indicate that this model could be suitable to detect human pro-carcinogens, in particular compounds bioactivated through CYP2E1 and 1A2. Nonetheless, more data on the characterization of xenobiotic metabolism in 3D HepaRG spheroids are required. Interestingly, several studies have shown that 3D models of PHH, HepaRG and HepG2 cells were more predictive than 2D for hepatotoxicity^24,36–40^. The major weakness for using the classical comet assay on the 3D HepaRG model is the low number of cells contained in one spheroid. In order to reduce the number of spheroids to pool and to improve both reproducibility and throughput, it could be worth adapting the CometChip Platform, which has been validated with 2D cultures, on 3D models^41^. A recent study also showed promising results for the micronucleus test using HepG2 3D models with B[a]P and PhIP^23^. Contrary to HepG2 spheroids, the absence of cell division in 3D HepaRG spheroids will need to be overcome to adapt the micronucleus assay.

In conclusion, we first report here the adaption of the comet assay in 3D HepaRG spheroids. We showed that 3D HepaRG spheroids are also an attractive model for *in vitro* genotoxicity testing and further experiments are required to prove that this model can improve the predictivity of *in vitro* models to detect human carcinogens.
